# Genetic, Reproductive and Hematological Toxicity Induced in Mice Exposed to Leachates from Petrol, Diesel and Kerosene Dispensing Sites

**DOI:** 10.5696/2156-9614-7.16.58

**Published:** 2017-12-18

**Authors:** Okunola A. Alabi, Babatunde E. Esan, Adewale A. Sorungbe

**Affiliations:** 1 Department of Biology, Federal University of Technology, Akure, Ondo State, Nigeria; 2 Department of Basic Sciences, Babcock University, Ilisan Remo, Ogun State, Nigeria

**Keywords:** petrol, diesel, kerosene, genotoxicity, hematology, DNA damage

## Abstract

**Background.:**

With a population of over 165,000,000, growing at an average rate of 2.7% per annum and an economic growth rate of about 5.7% in the past five years, the market for refined petroleum products in Nigeria is growing. As a result, the number of filling stations is increasing.

**Objectives.:**

The present study evaluated the reproductive and genetic toxicity of simulated leachate of soil from petrol, diesel and kerosene dispensing sites in a filling station using the murine sperm abnormality test, sperm count and bone marrow micronucleus assay.

**Methods.:**

Simulated leachate of soil collected from petrol, diesel and kerosene dispensing sites in a filling station was intraperitoneally administered to mice at different concentrations. Bone marrow micronucleus assay was carried out after 5-days exposure, while sperm morphology assay was carried out 35 days from the first day of exposure. Alterations to hematological parameters were evaluated and physico-chemical analysis of the leachate samples was also carried out.

**Results.:**

The results showed a significant (p<0.05) concentration-dependent increase in abnormal sperm cells and decrease in mean sperm count in all the samples tested. Increased induction of micronucleated polychromatic erythrocytes was observed in the exposed mice. Hematological analysis showed a significant (p<0.05) increase in the values of white blood cell count (WBC), lymphocytes, neutrophils, monocytes, eosinophils and mean corpuscular volume (MCV), while a significant (p<0.05) reduction in basophils, hemoglobin, mean corpuscular hemoglobin (MCH), packed cell volume and mean corpuscular hemoglobin concentration (MCHC) values were observed.

**Discussion.:**

In the present study, simulated leachates from soil obtained from petrol, diesel and kerosene dispensing sites were shown to cause genomic disruptions in germ and somatic cells, and hematotoxicity in an animal model. These observed reproductive, genetic and hemato-toxicities are believed to be caused by the presence of lead, copper, mercury, polycyclic aromatic hydrocarbons, and benzene in the samples.

**Conclusions.:**

This study showed the negative impact of petroleum products in the contamination of soil, with a capability of inducing genetic damage in somatic and germ cells of exposed plants and animals.

**Ethics Approval.:**

The study was approved by the ethical committee of the Federal University of Technology, Akure, Ondo State, Nigeria.

## Introduction

Petroleum products such as premium motor spirit (petrol), automated gas oil (diesel) and dual purpose kerosene are products utilized at an average of 60 million liters per day in Nigeria.^1^ Petroleum products are used as fuel in automobiles, generators, industrial plants and for cooking purposes, making petroleum an essential commodity that is needed for daily operations at the individual, industrial and national level.^2^ As a result of higher standards of living, the number of cars on the road is also on the increase in Nigeria. The need for a constant power supply to aid uninterrupted production of goods and services and for domestic use has led to increasing demand for fuel by Nigerians. With a population of over 165,000,000, growing at an average rate of 2.7% per annum and an economic growth rate of about 5.7% in the past five years, the market for refined petroleum products in Nigeria is growing. As a result, the number of filling stations where refined petroleum products are sold is increasing. Indiscriminate locating of filling stations is of public health concern, as these filling stations are located around residential and agricultural areas for easy accessibility to consumers, without regard to possible health impacts.

Petroleum products are common soil contaminants and often contain potentially toxic compounds, particularly toxic heavy metals, benzene, polycyclic aromatic hydrocarbons (PAHs) and polychlorinated biphenyls (PCBs).^3^ Constituents of petroleum products released into the environment can pose risks to ecosystems and human health. Some compounds in petroleum products are known to be mutagenic, genotoxic, carcinogenic, neurotoxic, hematotoxic and immunotoxic.^4–6^ Extensive chemical extraction and analysis of petroleum-contaminated soil can provide detailed information about total contaminant concentrations. However, the potential impact on the ecosystem may not be easily predicted using only concentration data.

Humans may be exposed to chemical constituents of petroleum products by breathing, eating, drinking or by skin contact. Workers using petroleum products may be exposed to higher levels of these chemical constituents through skin contact or by breathing contaminated air. Petroleum products can also enter the groundwater, thereby contaminating drinking water. Children may be exposed by playing in soil contaminated with petroleum products.

Benzene, toluene, and xylene (which are present in gasoline) have been reported to affect the human central nervous system.^3^ Breathing toluene at concentrations greater than 100 parts per million (100 ppm) for more than several hours can cause fatigue, headache, nausea, and drowsiness. Swallowing petroleum products such as gasoline and kerosene can cause irritation of the throat and stomach, central nervous system depression, difficulty breathing, and pneumonia from breathing liquid into the lungs. Benzene has been shown to cause cancer (leukemia) in humans and the International Agency for Research on Cancer (IARC) has determined that benzene is carcinogenic to humans (Group 1 classification). Other compounds or petroleum products such as benzo(a)pyrene and gasoline are considered to be probably or possibly carcinogenic to humans (IARC Groups 2A and 2B, respectively) based on cancer studies in people and animals. Most of the other total petroleum hydrocarbons compounds and products are considered not classifiable (Group 3) by the IARC.^3^

**Abbreviations**
*IARC*: International Agency for Research on Cancer
*MCH*: Mean corpuscular hemoglobin
*MCHC*: Mean corpuscular hemoglobin concentration
*MCV*: Mean corpuscular volume
*MNPCE*: Micronucleated polychromatic erythrocytes
*NCE*: Mormochromatic erythrocytes
*PCE*: Polychromatic erythrocytes
*RBC*: Red blood cell count
*WBC*: White blood cell count

The use of bioassays for ecotoxicity evaluation of contaminated soil has gained widespread attention over the past two decades. Since chemical analysis alone cannot adequately assess the potential ecological impact of contaminated soil, bioassays have proven to be good complements.^7^ Bioassays have been demonstrated to be important in assessing the effect of a complex mixture of compounds such as petroleum. Bioassays can also be useful in predicting the bioavailability of contaminants, since responses can occur at contaminant levels lower than those that can be easily detectable by chemical assays. Bioassays used for soil evaluation may involve direct exposure to the soil or leachates, raw or simulated, from the soil. Either method assumes that the organisms are being exposed to readily available contaminants.

Since petroleum is a major contaminant of terrestrial soils worldwide, it is important to use various bioassay species for public health risk assessment. The goal of the present study was to assess the possible genetic, reproductive and hematological toxicity of petrol-, diesel- and kerosene-contaminated soil in a filling station in Nigeria using murine bone marrow micronucleus assay, sperm morphology test, sperm count and analysis of hematological parameters.

## Methods

### Sampling Site

The study site was a filling station located on the Lagos-Sagamu expressway, Ogun State, southwest Nigeria. The filling station is divided into three areas, with each area selling either petrol (Site 1), diesel (Site 2) or kerosene (Site 3). It has been in operation for about 35 years and services an average of 1250 and 1065 vehicles per day with petrol and diesel, respectively, as well as 980 kerosene buyers (information obtained from the filling station records). The sites with diesel and kerosene spillage had a characteristic black color, while the site with petrol spillage had a characteristic brown color. The soil surfaces of the three sites were compact.

### Sample Collection and Simulation of Leachate

Soil samples were collected at each of the three sites using a sterile trowel after clearing debris from the soil surface and transferred directly into clean, sterile containers. Samples were transported to the laboratory, air-dried for 12 weeks and subsequently ground to powder. Leachate simulation from the soil was carried out according to the American Society for Testing and Materials category A extraction procedure as modified by Bakare et al.^8^ Aqueous extraction of the soil was favored in our study because it is the most appropriate extraction method for human exposure in the study area compared to organic extraction. A soil sample from an area without any history of petroleum product spillage in Ogun State was collected and used as a control for the physico-chemical analysis. The leachate samples were filtered using 15 cm filter paper (Whatman®, Maidstone, UK), pH measured and stored at 4° C until use.

### Determination of Physical and Chemical Parameters

For the determination of physical and chemical parameters, sulfate (SO_4_), nitrate (NO_3_), phosphate (PO_4_), ammonia (NH_3_), calcium (Ca), iron (Fe), magnesium (Mg) and manganese (Mn) were analyzed in the samples. The concentrations of some potentially genotoxic heavy metals cadmium (Cd), chromium (Cr), arsenic (As), nickel (Ni), copper (Cu), cobalt (Co), mercury (Hg), zinc (Zn) and lead (Pb) were analyzed using an atomic absorption spectrophotometer (Perkin Elmer A Analyst 200), and PAHs and benzene were measured according to standard analytical methods.^9–10^

### Animal Model

Young male Swiss albino mice (Mus musculus) were obtained from the Department of Physiology, University of Ibadan, Nigeria, and housed in polypropylene cages on paddy husk bedding. All the animals received standard laboratory food and water *ad libitum* during the experimental period. A two-week acclimatization period was observed before the start of the experiment. Animals were grouped according to weight at the start of the experimental period. Eight-week-old mice were used for the micronucleus and hematotoxicity tests, while 12- to 14-week-old mice were used for the sperm count and sperm morphology assay. Animal studies were performed in accordance with recommendations by the National Institutes of Health (NIH) guidelines for the care and use of animals (NIH publication, 1996 edition).^11^ The study was approved by the ethical committee of the Federal University of Technology, Akure, Ondo State, Nigeria.

#### Sperm Abnormality Assay

After the acute toxicity study (data not shown), five concentrations of 1, 5, 10, 25 and 50% (v/v, simulated leachate/distilled water) of each of the simulated leachates and five mice per concentration were utilized. The highest concentration utilized (50%) was lower than the lethal dose (LD_50_) level obtained in the preliminary acute toxicity study. Distilled water and cyclophosphamide (20 mg/kgbw) were used as negative and positive controls, respectively. The mice were injected intraperitoneally with 0.5 mL of each of the simulated leachates per day for 5 consecutive days and left for another 30 days after the last day of exposure without any treatment as spermatogenesis takes about 34.5 days to complete in mice.^12^ Hence, spermatogonia exposed at the first day of treatment would have completed spermatogenesis 35 days later. The intraperitoneal route was chosen because it is one of the fastest and most efficient routes for delivery of test samples into experimental animals.^13^ Animals were sacrificed after 35 days from the first exposure period by cervical dislocation and the testes were dissected. Both cauda epididymes were removed and placed in a watch glass containing 1 ml phosphate buffered saline (pH=7.2). The cauda epididymes were minced thoroughly and the suspension obtained was filtered through a fine mesh cloth to remove tissue debris and stained with 1% eosin Y for about 20 minutes. A drop of the sperm suspension was smeared on a clean slide. One thousand sperms per animal were scored from each group for the presence of sperm shape abnormalities following the criteria of Wyrobek et al.^14^

#### Sperm Count

The cauda epididymis was further minced in 1 mL of phosphate buffered saline (pH 7.4) and the obtained suspension was filtered through a fine mesh cloth to remove tissue debris. One drop of 1% eosin Y was added to the filtrate and left for 30 minutes. The stained sperm suspension was sucked slowly into a leukocyte hemocytometer exactly up to the 0.5 mark and then further diluted with phosphate buffered saline up to the 11 mark and mixed thoroughly. The diluted suspension was charged into a Neubauer counting chamber. The sperm count was performed according to the standard procedure.^15^ Briefly, the sperm in 8 squares, excluding the central erythrocyte area, were counted and the total count was then multiplied by 5 × 10^4^ to obtain total sperm per epididymis.

#### Bone Marrow Micronucleus Assay

Seven groups of mice (weight range: 22–30 g, 5 mice per group) per leachate sample at the same concentrations as the sperm morphology assay were utilized.

Distilled water and cyclophosphamide (20 mg/kgbw) were used as negative and positive controls, respectively. The mice were exposed daily by intraperitoneal injection for 4 consecutive days and then sacrificed by cervical dislocation on the 5th day. The bone marrow micronucleus assay was performed based on the method of Schmid.^16^,^17^ The femurs from each animal were removed and muscle was cleaned away from the bone. The proximal and distal ends of the femur were carefully shortened with scissors and the epiphyses removed until a small opening to the marrow lumen became visible. The femur was placed on the edge of a pre-labeled plastic centrifuge tube (1 animal per tube), which corresponded to the animal number. A syringe containing 2–3 mL of fetal bovine serum was used to flush bone marrow into the tube containing 2 mL of fetal bovine serum. Bone marrow cells were centrifuged at 1000 rpm for 5 minutes (Eppendorf, Model 5810). After centrifugation, the supernatant was removed and 400 μL fresh fetal bovine serum was added to re-suspend the pellet. A volume of 10 μL of the final cell suspension was put on a clean slide labeled with the study and animal number. Cells were spread over the slide by pulling the material across the slide using the edge of a second clean slide. Slides were air dried at room temperature, fixed for 10 minutes in absolute methanol and then air-dried. Slides were stained with May-Grunwald and Giemsa stains. Five slides were prepared per animal and at least 1000 cells per slide were scored at ×1000 for micronucleated polychromatic erythrocytes (MNPCE) and normochromatic erythrocytes (NCE) (total of 5000 cells per animal).

#### Hematological Analysis

After sacrifice, the blood of each mouse was collected into an ethylenediaminetetraacetic acid specimen bottle by cardiac puncture.

All samples were stored in a refrigerator at 4° C until the time of analysis. The hematological values including white blood cell count (WBC), red blood cell count (RBC), hemoglobin concentration, mean corpuscular volume (MCV), mean corpuscular hemoglobin (MCH), mean corpuscular hemoglobin concentration (MCHC) and differential white blood cell count including lymphocytes, monocytes, neutrophils, and eosinophils were determined using an automatic blood analyzer (Swelab Alfa, Biozen, Sweden).

### Statistical Analysis

The SPSS® 15.0 and Microsoft® Excel 2003 statistical packages were used for the data analysis. Data obtained were expressed as % frequency and mean ± standard deviation in each assay. Percentage polychromatic erythrocytes (PCE), NCE, MNPCE, and micronucleated normochromatic erythrocytes were calculated and the ratio of PCE to NCE was also recorded. Significance at the different dose-level of each assay was tested using Dunnett's t-test. Differences between the negative control-group and individual dose-groups were analyzed at the 0.05 probability levels. The significance of differences among the mean sperm counts of different groups was determined by Student's t-test.

## Results

### Physicochemical Analysis

Table 1 shows the results of the physicochemical and heavy metal characteristics of simulated leachates of soil from petrol (Site 1), diesel (Site 2) and kerosene (Site 3) dispensing sites at a petrol filling station. The pH of the petrol dispensing soil was 9.5, which is higher than the maximum allowable limit by standards organizations.^18^,^19^ Only nitrate was within the allowable limits at all three sites. The concentrations of PO_4_, NH_3_, SO_4_, Cd, Pd, Cu, Fe, Zn, Hg, Mn, Cr, Ni, Co and As were higher than the maximum allowable limits in all three sites. Generally, soil from the petrol dispensing site had higher concentrations of each of the physico-chemical parameters, followed by the soil from the diesel dispensing site. Soil from the kerosene dispensing site had the lowest concentration of the parameters. A significant level of PAHs was recorded in the three sites with diesel, petrol and kerosene dispensing soils having 4.42, 3.25 and 2.21 mg/kg dw, respectively. Benzene measured at 1.26, 1.01 and 1.11 mg/kg dw for petrol, diesel and kerosene soils, respectively.

**Table 1 —:**
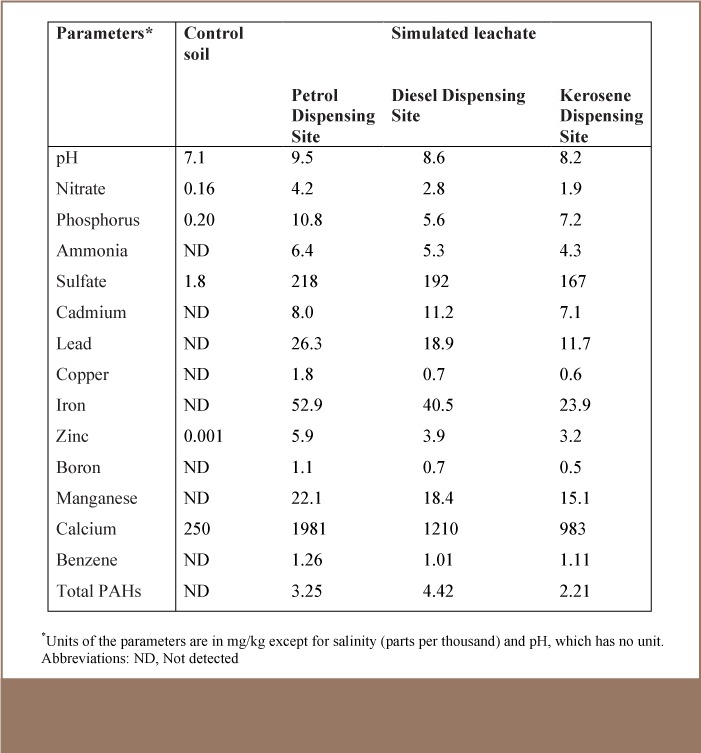
Physicochemical, Heavy Metals and Organic Characteristics of Petrol, Diesel and Kerosene Dispensing Site Soil Simulated Leachate at a Petrol Filling Station

### Sperm Abnormality Assay

**Figure 1 —:**
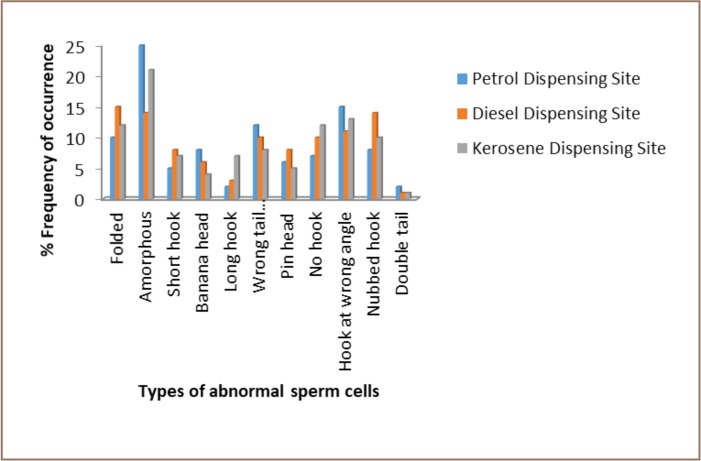
Percentage frequencies of different types of sperm abnormalities observed in mice treated with different concentrations of simulated leachates from soil of petrol, diesel and kerosene dispensing sites at a petrol filling station

In the sperm abnormality assay, sperm morphology and sperm counts were studied. Several morphological changes were observed such as amorphous head, hook-less sperm, folded type sperm, hook at wrong angle, knobbed hook, double tail and banana-shaped sperms. Figure 1 shows the types of abnormal sperm cells and the percentage frequency of each abnormality type. Table 2 shows the percentage frequency of abnormal sperm cells induced in mice exposed to leachates from soil of the petrol, diesel and kerosene dispensing site after 35 days. There was an increase in morphological abnormalities in leachate-treated animals in a concentration-dependent manner in the three sites. In the leachate of soil from the petrol dispensing site, there was a statistically significant (p<0.05) increase in abnormality at a 5% concentration and above, while significance was recorded at 10% concentration and above for leachates of soil from both diesel and kerosene dispensing sites compared with the negative control. Generally, soil from the petrol dispensing site induced a higher frequency of abnormal sperm cells followed by soils from diesel and kerosene dispensing sites (*Table 2*).

**Table 2 —:**
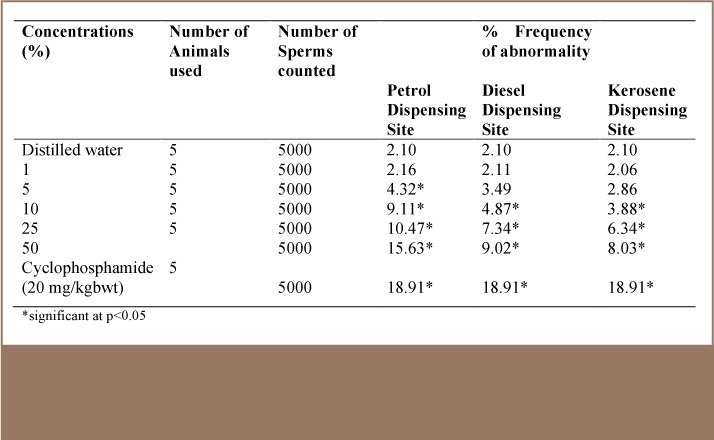
Summary of Morphologically Abnormal Sperm Cells Induced in Mice After 5 Weeks Exposure to Different Concentrations of Petrol, Diesel and Kerosene Dispensing Site Soil Simulated Leachate at a Petrol Filling Station

With regard to sperm count, the number of sperm per epididymis decreased in the leachate-treated animals in a concentration-dependent manner. Table 3 shows the mean sperm count of mice exposed to different concentrations of simulated leachates from soil of petrol, diesel and kerosene dispensing sites. The mean sperm count in the negative control was 1.21 × 10^8^. There was a significant (p<0.05) decrease in the mean sperm count at a 5% concentration and above in the soil of petrol dispensing site, while a significant decrease was recorded at 25 and 50% concentrations in soil from diesel and kerosene dispensing sites. Similar to the sperm morphology assay, soil from the petrol dispensing site induced the most significant decrease in mean sperm count, followed by soil of diesel and kerosene dispensing sites.

**Table 3 —:**
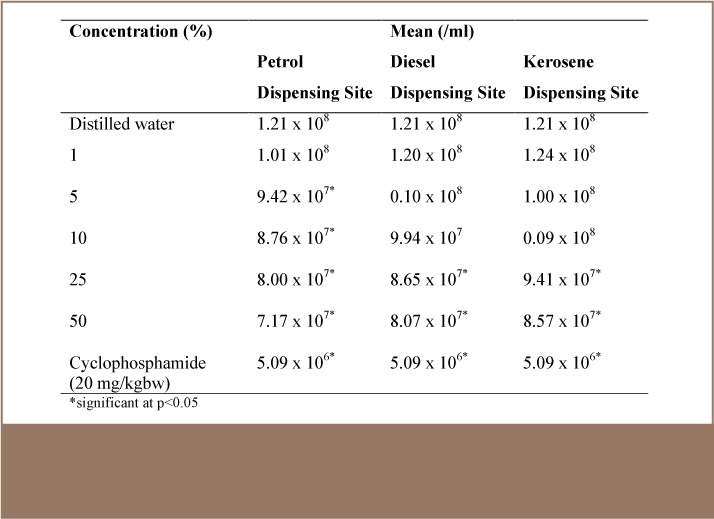
Mean (mL) Sperm Count of Mice Exposed to the Different Concentrations of Petrol, Diesel and Kerosene Dispensing Site Soil Simulated Leachate at a Petrol Filling Station

### Bone Marrow Micronucleus Assay

**Figure 2 —:**
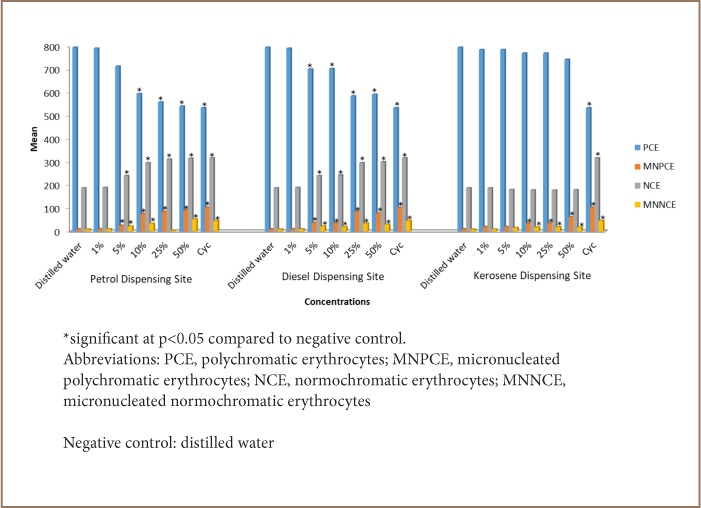
Mean PCE, MNPCE, NCE and MNNCE induced in the bone marrow of mice exposed to different concentrations of simulated leachates from soil of petrol, diesel and kerosene dispensing sites at a petrol filling station

The leachates induced micronucleus in a concentration-dependent manner in the bone marrow of exposed mice. The results of micronucleus induction are summarized in Figure 2. A significant (p<0.05) increase of MNPCE frequencies in bone marrow was observed in mice exposed to leachates of soil from petrol and diesel dispensing sites at a 5% concentration and above compared to the control group, while leachate of soil from the kerosene dispensing site induced significant micronucleus at a 10% concentration and above. A similar trend was observed with the micronucleated normochromatic erythrocytes in the three sites. The ratio of PCE to NCE decreased significantly (p<0.05) from 6.72 in the negative control group to 1.48 at a 50% concentration of leachate of soil from the petrol dispensing site.

### Hematological Analysis

The leachates markedly affected the hematological profile of the test animals. Generally, in comparison to control mice, the values of WBC, lymphocytes, neutrophils, monocytes, eosinophils and MCV increased significantly (p<0.05), while a significant (p<0.05) reduction in basophils, hemoglobin, MCH, packed cell volume and MCHC values was observed. There was, however, no significant (p>0.05) reduction in RBC values in the three leachate samples (*Tables 4, 5, 6*). In most of the parameters, significant changes were observed in the leachate from the kerosene dispensing site at concentrations of 10% and above (*Table 6*). However, in leachates from the petrol and diesel dispensing sites, significant changes were observed at concentrations of 5% and above (*Tables 5 and 6*).

**Table 4 —:**
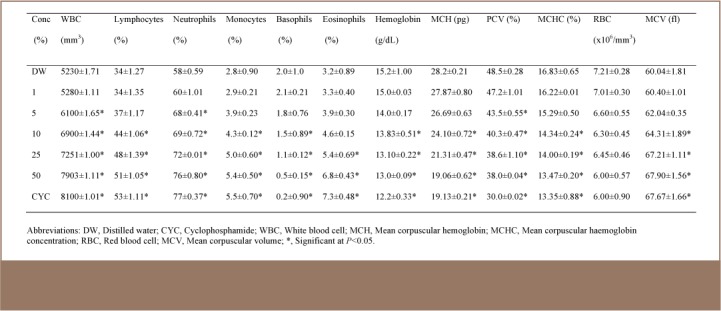
Hematological Parameters of Mice Exposed to Different Concentrations of Petrol Dispensing Site Soil Simulated Leachate at a Petrol Filling Station

**Table 5 —:**
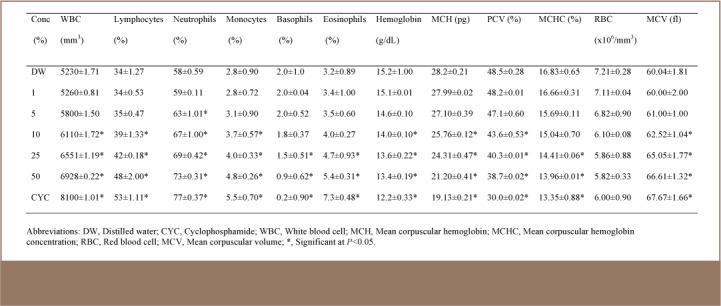
Hematological Parameters of Mice Exposed to Different Concentrations of Diesel Dispensing Site Soil Simulated Leachate at a Petrol Filling Station

**Table 6 —:**
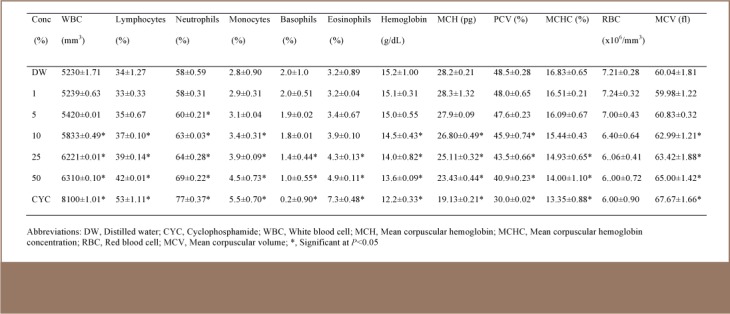
Hematological Parameters of Mice Exposed to Different Concentrations of Kerosene Dispensing Site Soil Simulated Leachate at a Petrol Filling Station

## Discussion

Spillages of petrol, diesel and kerosene are common occurrences during self-refueling using hoses and kegs at filling stations. Contamination of soil with petroleum products is an increasingly frequent ecological problem. Accumulation of these spillages over the years can make the soil of filling stations a public health concern, not only to customers of the filling stations, but to workers and residents in their vicinity. The wide variety of chemicals involved and the presence of metabolites formed during biodegradation of hydrocarbons from these petroleum products make them hazardous. Some of the metabolites may be characterized by a higher toxicity compared with the initial substrate; others may exhibit mutagenic or carcinogenic activity.^20^,^21^ In the present study, simulated leachates from soil obtained from petrol, diesel and kerosene dispensing sites were shown to cause genomic disruptions in germ and somatic cells, and hematotoxicity in an animal model.

The results of the sperm morphology assay showed that the leachates from the three soils induced significant abnormal sperm cells with a concurrent significant reduction in mean sperm count in exposed mice. An important aspect in sperm quality assessment, which is also a key index in reproductive toxicity and mutagenicity of exogenous chemicals evaluation, is sperm morphology. Spermatogenesis is temporally and spatially a highly regulated differentiating system.

Due to rapid cell division, germ cells, especially differentiating spermatozoa, are extremely susceptible to cytotoxic agents. The non-proliferating Leydig cells and Sertoli cells survive most cytotoxic therapies, but could suffer functional damage.^22^ For assessment of the genotoxic potential of chemicals, sperm head abnormality is the most reliable short term biological indicator.^23^ Sperm head morphology gives an indication of spermatozoa's functional capability and reveals the quality of the sperm DNA (deoxyribonucleic acid).^24^ Where there are point mutations or other chromosome variations as a result of exposure to genotoxic agents, sperm abnormalities are the resultant end points.^25^ Sperm head abnormalities are most likely a reflection of a change in DNA content.^26^ Sperm head abnormalities may be a result of small endoplasmic deletions or point mutations or testicular DNA alteration which subsequently leads to disruptions of spermatozoa differentiation.^27^ The morphological abnormalities of sperm cells might be due to the damage exerted on differentiated spermatogonia, or the interface between differentiated spermatogonia and spermatocyte. It is a general belief that differentiated spermatogonia are the most sensitive spermatogenic cellular type to the action of various chemical agents in the production of abnormal sperms.^28^ The simulated leachates in this study therefore contained constituents capable of altering the production as well as the process of spermatogenesis. Sperm function is strictly correlated with sperm morphology and sperm motility is usually the best predictor of fertility potential in men.^29^ This study suggests that there might be potential fertility problems in men employed at filling stations who are constantly exposed to petroleum products.

Germ line mutation may not be necessarily responsible for the elevated levels of sperm head abnormalities in both animal models and humans following exposure to a physical and chemical mutagen according to Wyrobek and Bruce.^30^ There is also the possibility of somatic alterations in the animal which might lead to increased sperm abnormalities. This study further reports a significant increase in genetic damage in the bone marrow cells (somatic cells) of exposed mice with potential correlation with observed sperm abnormalities.

The study of the clastogenic and aneugenic effects of leachates from soil of petrol, diesel and kerosene dispensing sites using micronucleus assay revealed a significant induction of micronucleus in the bone marrow of exposed mice compared to the negative control group. Micronuclei are cytoplasmic chromatin masses with the appearance of small nuclei, which are produced due to chromosome lagging at anaphase or as a result of acentric chromosomal fragments. They provide a quantifiable measure of recent DNA injury resulting when acentric fragments or whole chromosomes are left behind from the main nucleus at telophase. Micronuclei can be induced by defects in the cell repair machinery and accumulation of DNA damages and chromosomal aberrations. A variety of genotoxic agents may induce micronucleus formation leading to cell death, genomic instability, or cancer development. An increase in the frequency of micronucleated polychromatic erythrocytes in treated mice is an indication of induced chromosome damage or damage to the mitotic apparatus of erythroblasts. Whole chromatids or chromosomes in micronucleus are formed due to deficiencies in chromosome segregation during anaphase usually caused by mitotic spindle failure, kinetochore damage, centromeric DNA hypomethylation, and defects in the cell cycle control system.^31^

The present study also showed a significant decrease in PCE/NCE ratio in treated mice compared to the control group. The PCE/NCE ratio is an indicator of the acceleration or inhibition of erythropoiesis and varies with scoring interval. A continuous decline in the PCE/NCE ratio may reflect the inhibition of cell division, the killing of erythroblasts, the removal of damaged cells, or dilution of the existing cell pool with newly formed cells.^32^

Irrespective of the route of exposure, the blood stream is usually involved in the movement of exogenous chemical agents around the body. This has made hematological parameters particularly good indicators of toxicity which can be used to establish the effect of an agent on the well-being of animals and humans. The hematopoietic system has been a very important index for the assessment of the physiological and pathological state of mammals because of its sensitivity to xenobiotics. The result of the hematological parameter analysis of treated mice in this study showed an adverse alteration of the assessed profile.

The concentration of WBC in the plasma is an indicator of an organism's defensive potential against infections. The body uses lymphocytes (eosinophils) for protection against allergic reactions and parasites. Therefore, increased levels of WBC and lymphocytes in this study may indicate an allergic response of the treated mice to the constituents of the simulated leachates. Red blood cell indices, particularly MCV, MCH and MCHC, are very important in the morphological classification of anaemias.^33^ Mean corpuscular volume is the average volume of red cells in a specimen. The increase in MCV observed in this study is an indication of macrocytic anemia. Indeed, studies in experimental animals, petrol attendants and auto mechanics have shown that anemia is induced with exposure to petroleum products.^34^,^35^ The leachates in this study further caused reduced MCHC, a hypochromic condition. Mean corpuscular hemoglobin concentration is the average concentration of hemoglobin in the RBCs which is contained within a specific sample. This study also reported decreased packed cell volume and hemoglobin with increased WBC, which is similar to the observations of Uboh et al., where inhalational exposure to petrol vapors caused decreased packed cell volume, hemoglobin, and RBC count, as well as increased WBC counts in Wister rats.^36^

The reported genomic disruptions and hematotoxicity in the present study are believed to be due to the chemical constituents of the leachate. The results of the physico-chemical and organic analysis showed the presence of heavy metals, benzene and PAHs, some of which were at higher concentrations than the maximum allowable limits set by regulatory organizations. Some of these constituents have been reported to cause DNA damage and adversely alter the hematological profile of exposed organisms.^37–39^

Similar to the findings in the present study, the presence of significant concentrations of heavy metal and organics from contaminated soil leachates have been reported in Nigeria. Studies by Sanusi, Oyiboka and Oluseyi et al. documented heavy metal contamination of underground water from landfill leachate.^40–42^ Bakare et al. and Alabi et al. reported heavy metal and organic contamination of underground water by leachate of contaminated soils.^43^,^39^

There is evidence that heavy metals affect cellular organelles and components including mitochondrial, endoplasmic reticulum, cell membrane, lysosome, nuclei and certain enzymes required for damage repair, detoxification and metabolism in biological systems.^44^ Heavy metals have been reported to interact with DNA and nuclear protein to cause DNA damage and conformational changes which might lead to carcinogenesis, apoptosis or cell cycle modulation.^45^,^46^ In addition, As, Cd, Cr, Pb and Hg have been reported to be carcinogenic and genotoxic.^47–52^

The mechanisms of heavy metals toxicity have been reviewed.^53^ Lead toxicity mainly occurs due to the ability of Pb metal ions to replace other bivalent cations such as calcium ions (Ca2+), magnesium ion (Mg2+), ferrous ion (Fe2+) and monovalent cations like sodium ion (Na+), which ultimately disturbs the biological metabolism of the cell. These ionic mechanisms cause significant alterations in biological processes such as cell adhesion, intra- and inter-cellular signaling, protein folding, maturation, apoptosis, ionic transportation, enzyme regulation, and release of neurotransmitters.^54^ Mercury can cause disruption to the membrane potential, interrupt intracellular calcium homeostasis, damage the tertiary and quaternary protein structure and alter cellular function. It can also intervene with the process of transcription and translation, resulting in the disappearance of ribosomes and the eradication of endoplasmic reticulum and the activity of natural killer cells. Cellular integrity is also affected, causing free radical formation.^53^,^55^ The mechanism of Cd toxicity is not yet clearly understood, but its effects on cells are known.^56^ However, Cd has the capability to bind with cysteine, glutamate, histidine and aspartate ligands and can lead to iron deficiency.^57^ Hexavalent chromium (Cr(VI)) has been found to be much more dangerous than trivalent chromium (Cr(III)) because it enters the cells more readily than Cr(III). Due to its mutagenic properties, Cr(VI) has been categorized as a group 1 human carcinogen by the IARC.^58^,^59^ Iron has been known to disrupt oxidative phosphorylation and also cause lipid peroxidation, which results in severe damage to mitochondria, microsomes and other cellular organelles.^60^ Iron has been shown to produce hydrogen free radicals which attack DNA, resulting in cellular damage, mutation and malignant transformations, which in turn can cause an array of diseases.^61^

Polycyclic aromatic hydrocarbons have been shown to be genotoxic to humans, and are potential carcinogenic agents.^62–64^ The United States Environmental Protection Agency has designated 16 PAHs as priority pollutants.^65^ This classification considers the risk to health and the degree of carcinogenic and mutagenic potential. In addition, benzene has been reported to increase the risk of several acute and chronic diseases such as acute myeloid leukemia, acute and chronic lymphocytic leukemia, non-Hodgkin lymphoma, multiple myeloma and aplastic anemia in humans.^66^ Indeed, mutagenic, genotoxic, carcinogenic, neurotoxic, immunotoxic and hemotoxic effects of petroleum and petrochemical products' constituents have been reported in experimental studies on humans, animals and prokaryotes.^4–6^,^67^

## Conclusions

In conclusion, the present study reports genetic toxicity in somatic cells, reproductive toxicity and hematological alterations in mice exposed to simulated leachate of soil from petrol, diesel and kerosene dispensing sites around a filling station. High concentrations of heavy metals and organic pollutants in these samples are believed to be responsible for these observed toxicities. The observed adverse effects of this type of pollutant is an indication of possible health impacts to the exposed general populace.
